# Ethyl 2-(4-hydr­oxy-1-methyl-2-oxo-1,2-dihydro­quinolin-3-yl)acetate

**DOI:** 10.1107/S1600536809011921

**Published:** 2009-04-08

**Authors:** Igor V. Ukrainets, Svetlana V. Shishkina, Oleg V. Shishkin, Alexandra A. Davidenko, Andrei A. Tkach

**Affiliations:** a National University of Pharmacy, 4 Blyukhera ave., Kharkiv 61002, Ukraine; b STC "Institute for Single Crystals", National Academy of Sciences of Ukraine, 60 Lenina ave., Kharkiv 61001, Ukraine

## Abstract

In the title compound, C_14_H_15_NO_4_, the bicyclic fragment and the ester group form a dihedral angle of 86.7 (2)°. Inter­molecular O—H⋯O and C—H⋯O hydrogen bonding connects mol­ecules into a helix along the crystallographic *b* axis.

## Related literature

For esters of 4-hydr­oxy-2-oxo-1,2-dihydro­quinolin-3-acetic acids as non-steroidal anti-inflammatory drugs, see: Ukrainets *et al.* (2001 ▶). For their use in the synthesis of natural alkaloids, see: Ramesh & Shanmugam (1985 ▶); Geismann & Cho (1959 ▶) and in highly active anti­thyroid substances, see: Ukrainets *et al.* (1997 ▶). For van der Waals radii, see: Zefirov (1997 ▶). For related structures, see: Jurd *et al.* (1983 ▶); Ukrainets *et al.* (2000 ▶). For bond-length data, see: Bürgi & Dunitz (1994 ▶).
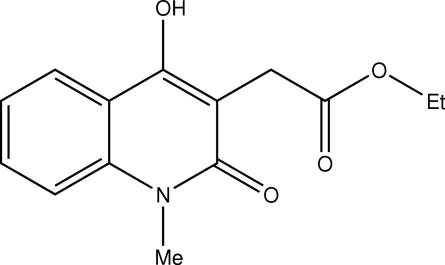


## Experimental

### 

#### Crystal data

C_14_H_15_NO_4_
*M* *_r_* = 261.27
Monoclinic, 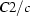

*a* = 21.608 (2) Å
*b* = 9.2155 (9) Å
*c* = 14.6795 (12) Å
β = 119.632 (9)°
*V* = 2540.8 (4) Å^3^
*Z* = 8
Mo *K*α radiation
μ = 0.10 mm^−1^
*T* = 293 K
0.30 × 0.30 × 0.20 mm

#### Data collection

Oxford Diffraction Xcalibur3 diffractometer
Absorption correction: none
13114 measured reflections
2862 independent reflections
2376 reflections with *I* > 2σ(*I*)
*R*_int_ = 0.030

#### Refinement

*R*[*F* ^2^ > 2σ(*F* ^2^)] = 0.060
*wR*(*F* ^2^) = 0.171
*S* = 1.19
2862 reflections
232 parameters
All H-atom parameters refined
Δρ_max_ = 0.28 e Å^−3^
Δρ_min_ = −0.19 e Å^−3^

### 

Data collection: *CrysAlis CCD* (Oxford Diffraction, 2005 ▶); cell refinement: *CrysAlis RED* (Oxford Diffraction, 2005 ▶); data reduction: *CrysAlis RED*; program(s) used to solve structure: *SHELXTL* (Sheldrick, 2008 ▶); program(s) used to refine structure: *SHELXTL*; molecular graphics: *XP* (Siemens, 1998 ▶); software used to prepare material for publication: *SHELXTL*.

## Supplementary Material

Crystal structure: contains datablocks I, global. DOI: 10.1107/S1600536809011921/kp2212sup1.cif

Structure factors: contains datablocks I. DOI: 10.1107/S1600536809011921/kp2212Isup2.hkl

Additional supplementary materials:  crystallographic information; 3D view; checkCIF report

## Figures and Tables

**Table 1:** Hydrogen-bond geometry (Å, °)

*D*—H⋯*A*	*D*—H	H⋯*A*	*D*⋯*A*	*D*—H⋯*A*
O2—H2*O*⋯O1^i^	0.95 (3)	1.71 (3)	2.649 (2)	169 (2)
C10—H10a⋯O1^i^	0.94 (2)	2.34 (3)	3.235 (2)	159 (2)

Symmetry code: (i) 
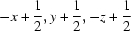
.

**Fig. 1.**
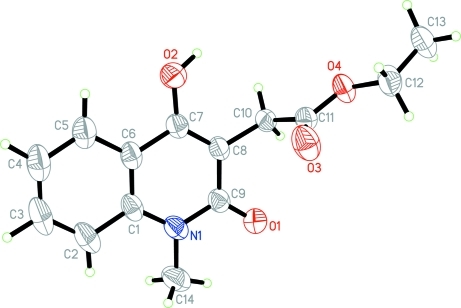
View of the title compound with atomic numbering. All atoms are shown with displacement ellipsoids drawn at the 50% probability level.

**Fig. 2.**
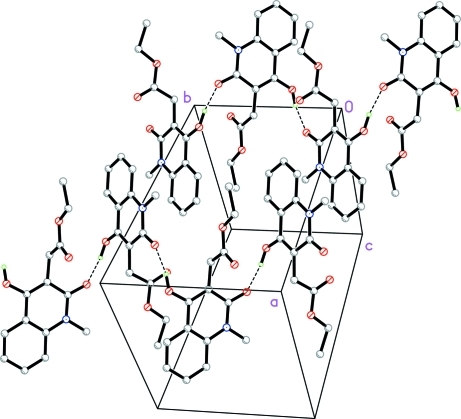
The packing of the molecules in a crystal. The hydrogen bonds are shown by dashed lines.

## supplementary crystallographic information

### Comment

Esters of 4-hydroxy-2-oxo-1,2-dihydroquinolin-3-acetic acids can be considered as non-steroid anti-inflammatory drugs (Ukrainets *et al.*, 2001). However they are of great interest for synthesis of natural alkaloids (Ramesh & Shanmugam, 1985; Geismann & Cho, 1959) and highly active antithyroid substances (Ukrainets *et al.*, 1997). In the present paper, we report the crystal structure of the (4-hydroxy-1-methyl-2-oxo-1,2-dihydro- quinolin-3-yl)-acetic acid ethyl ester (I) (Fig. 1). The bicyclic fragment and the C14, O1, C10 and O2 atoms are coplanar within 0.02 Å. The planar ester group at the C10 atom has orthogonal orientation with respect to the plane of quinolone bicycle (the C7—C8—C10—C11 torsion angle is 90.8 (2) %A) whereas the C8—C10—C11—O3 torsion angle is 7.3 (3) %A). The C9—O1 bond (1.250 (2) Å) is elongated as compared with its mean value (1.210 Å; Bürgi & Dunitz, 1994) owing to the formation of the intermolecular hydrogen bond O2—H2O···O1' (Table 1). The presence of hydrogen bond affects the orientation of the hydrogen atom of hydroxy group despite of strong repulsion with hydrogen atom of neighbouring methylene group: distance H10*a*···H2O is 2.09 Å [the van der Waals radii sum is 2.34 Å (Zefirov, 1997)]. It should be noted that the C7—O2 bond length (1.341 (2) Å) is close to its mean value 1.333 Å observed in earlier investigated compounds (Jurd *et al.*,  1983; Ukrainets *et al.*, 2000). In the crystal the molecules form the infinite helix along the [0 1 0] direction (Fig. 2) *via* the O2—H2O···O1 intermolecular hydrogen bond between hydroxyl group of one molecule and carbonyl group of quinolone fragment of neighbouring molecule. The C10—H10*a*···O1' intermolecular hydrogen bond (Table 1) occurs as well.

### Experimental

(4-Hydroxy-1-methyl-2-oxo-1,2-dihydroquinolin-3-yl)-acetic acid is synthesized from the methyl *N*-methyl anthranilate using the known method (Geismann & Cho, 1959) and then is esterified by ethanol (Ukrainets *et al.*, 2001). Yield 96%. *M*.p. 454–457 K.

### Refinement

All hydrogen atoms were located from electron density difference maps and were refined isotropically.

### Crystal data

**Table:**

C_14_H_15_NO_4_	*D*_x_ = 1.366 Mg m^−^^3^
*M**_r_* = 261.27	Melting point: 455 K
Monoclinic, *C*2/*c*	Mo *K*α radiation, λ = 0.71073 Å
*a* = 21.608 (2) Å	Cell parameters from 8736 reflections
*b* = 9.2155 (9) Å	θ = 4–32°
*c* = 14.6795 (12) Å	µ = 0.10 mm^−^^1^
β = 119.632 (9)°	*T* = 293 K
*V* = 2540.8 (4) Å^3^	Block, colourless
*Z* = 8	0.30 × 0.30 × 0.20 mm
*F*(000) = 1104	

### Data collection

**Table:**

Oxford Diffraction Xcalibur3 diffractometer	2376 reflections with *I* > 2σ(*I*)
Radiation source: Enhance (Mo) X-ray Source	*R*_int_ = 0.030
graphite	θ_max_ = 27.5°, θ_min_ = 3.2°
Detector resolution: 16.1827 pixels mm^-1^	*h* = −28→28
ω scans	*k* = −11→11
13114 measured reflections	*l* = −19→19
2862 independent reflections	

### Refinement

**Table:**

Refinement on *F*^2^	Primary atom site location: structure-invariant direct methods
Least-squares matrix: full	Secondary atom site location: difference Fourier map
*R*[*F*^2^ > 2σ(*F*^2^)] = 0.060	Hydrogen site location: difference Fourier map
*wR*(*F*^2^) = 0.171	All H-atom parameters refined
*S* = 1.19	*w* = 1/[σ^2^(*F*_o_^2^) + (0.0943*P*)^2^] where *P* = (*F*_o_^2^ + 2*F*_c_^2^)/3
2862 reflections	(Δ/σ)_max_ < 0.001
232 parameters	Δρ_max_ = 0.28 e Å^−^^3^
0 restraints	Δρ_min_ = −0.19 e Å^−^^3^

### Special details

**Table:**

Geometry. All e.s.d.'s (except the e.s.d. in the dihedral angle between two l.s. planes) are estimated using the full covariance matrix. The cell e.s.d.'s are taken into account individually in the estimation of e.s.d.'s in distances, angles and torsion angles; correlations between e.s.d.'s in cell parameters are only used when they are defined by crystal symmetry. An approximate (isotropic) treatment of cell e.s.d.'s is used for estimating e.s.d.'s involving l.s. planes.
Refinement. Refinement of *F*^2^ against ALL reflections. The weighted *R*-factor *wR* and goodness of fit *S* are based on *F*^2^, conventional *R*-factors *R* are based on *F*, with *F* set to zero for negative *F*^2^. The threshold expression of *F*^2^ > σ(*F*^2^) is used only for calculating *R*-factors(gt) *etc*. and is not relevant to the choice of reflections for refinement. *R*-factors based on *F*^2^ are statistically about twice as large as those based on *F*, and *R*- factors based on ALL data will be even larger.

### Fractional atomic coordinates and isotropic or equivalent isotropic displacement parameters (Å^2^)

**Table:**

	*x*	*y*	*z*	*U*_iso_*/*U*_eq_	
N1	0.12615 (8)	0.48562 (16)	0.00279 (10)	0.0447 (4)	
O1	0.23317 (6)	0.38519 (13)	0.11389 (9)	0.0511 (4)	
O2	0.14804 (7)	0.78108 (14)	0.22763 (10)	0.0528 (4)	
H2O	0.1932 (14)	0.807 (3)	0.284 (2)	0.086 (8)*	
O3	0.21567 (7)	0.39416 (16)	0.34316 (11)	0.0651 (4)	
O4	0.32312 (6)	0.48690 (14)	0.45168 (9)	0.0525 (4)	
C1	0.07158 (8)	0.58298 (18)	−0.01697 (12)	0.0434 (4)	
C2	0.00694 (10)	0.5816 (2)	−0.11336 (15)	0.0566 (5)	
H2	0.0020 (13)	0.511 (3)	−0.161 (2)	0.077 (7)*	
C3	−0.04624 (10)	0.6786 (3)	−0.13061 (17)	0.0652 (6)	
H3	−0.0931 (15)	0.677 (3)	−0.195 (2)	0.102 (9)*	
C4	−0.03752 (10)	0.7799 (3)	−0.05606 (18)	0.0638 (6)	
H4	−0.0737 (14)	0.850 (3)	−0.064 (2)	0.087 (7)*	
C5	0.02492 (9)	0.7826 (2)	0.03876 (16)	0.0541 (5)	
H5	0.0334 (10)	0.852 (2)	0.0948 (15)	0.054 (5)*	
C6	0.07974 (8)	0.68292 (17)	0.05940 (13)	0.0419 (4)	
C7	0.14509 (8)	0.68166 (17)	0.15888 (12)	0.0393 (4)	
C8	0.19716 (8)	0.58370 (17)	0.17776 (12)	0.0390 (4)	
C9	0.18749 (9)	0.47940 (18)	0.09900 (12)	0.0407 (4)	
C10	0.26583 (8)	0.57643 (19)	0.28059 (12)	0.0413 (4)	
H10B	0.3055 (9)	0.5445 (19)	0.2663 (13)	0.042 (4)*	
H10A	0.2774 (9)	0.668 (2)	0.3117 (14)	0.044 (5)*	
C11	0.26352 (8)	0.47532 (18)	0.35929 (13)	0.0416 (4)	
C12	0.32854 (11)	0.3976 (2)	0.53692 (15)	0.0564 (5)	
H12B	0.2882 (13)	0.416 (3)	0.5450 (19)	0.082 (7)*	
H12A	0.3291 (12)	0.291 (3)	0.5182 (18)	0.084 (7)*	
C13	0.39738 (15)	0.4393 (3)	0.63283 (17)	0.0757 (7)	
H13C	0.3974 (17)	0.546 (4)	0.658 (2)	0.132 (12)*	
H13B	0.4378 (17)	0.416 (4)	0.623 (3)	0.119 (11)*	
H13A	0.4017 (15)	0.380 (3)	0.688 (2)	0.105 (9)*	
C14	0.11943 (14)	0.3847 (3)	−0.07860 (17)	0.0627 (5)	
H14C	0.1113 (13)	0.444 (3)	−0.1404 (19)	0.084 (7)*	
H14B	0.1627 (16)	0.332 (3)	−0.050 (2)	0.105 (10)*	
H14A	0.0770 (14)	0.324 (3)	−0.0999 (19)	0.080 (7)*	

### Atomic displacement parameters (Å^2^)

**Table:**

	*U*^11^	*U*^22^	*U*^33^	*U*^12^	*U*^13^	*U*^23^
N1	0.0472 (8)	0.0479 (8)	0.0323 (7)	−0.0083 (6)	0.0145 (6)	0.0011 (6)
O1	0.0542 (7)	0.0477 (7)	0.0450 (7)	0.0045 (5)	0.0196 (6)	0.0028 (5)
O2	0.0436 (7)	0.0519 (7)	0.0528 (7)	−0.0003 (5)	0.0160 (6)	−0.0072 (6)
O3	0.0505 (8)	0.0751 (10)	0.0550 (8)	−0.0127 (6)	0.0149 (6)	0.0126 (7)
O4	0.0458 (7)	0.0627 (8)	0.0352 (6)	0.0027 (5)	0.0095 (5)	0.0072 (5)
C1	0.0368 (8)	0.0472 (9)	0.0359 (8)	−0.0093 (7)	0.0101 (7)	0.0112 (7)
C2	0.0476 (10)	0.0667 (13)	0.0378 (9)	−0.0157 (9)	0.0075 (8)	0.0124 (9)
C3	0.0387 (10)	0.0794 (15)	0.0531 (11)	−0.0089 (9)	0.0040 (8)	0.0275 (11)
C4	0.0373 (10)	0.0654 (13)	0.0707 (13)	0.0015 (9)	0.0129 (9)	0.0215 (11)
C5	0.0400 (9)	0.0511 (11)	0.0597 (11)	−0.0004 (8)	0.0159 (8)	0.0127 (9)
C6	0.0345 (8)	0.0410 (9)	0.0417 (8)	−0.0051 (6)	0.0123 (7)	0.0121 (7)
C7	0.0356 (8)	0.0383 (8)	0.0380 (8)	−0.0068 (6)	0.0137 (6)	0.0032 (6)
C8	0.0356 (8)	0.0384 (8)	0.0337 (8)	−0.0037 (6)	0.0100 (6)	0.0046 (6)
C9	0.0420 (9)	0.0398 (8)	0.0356 (8)	−0.0045 (6)	0.0156 (7)	0.0050 (6)
C10	0.0340 (8)	0.0394 (9)	0.0380 (9)	−0.0014 (6)	0.0084 (7)	−0.0008 (6)
C11	0.0365 (8)	0.0442 (9)	0.0372 (8)	0.0059 (6)	0.0128 (7)	−0.0017 (6)
C12	0.0600 (12)	0.0693 (14)	0.0412 (10)	0.0198 (9)	0.0259 (9)	0.0129 (9)
C13	0.0800 (17)	0.0909 (19)	0.0360 (10)	0.0189 (14)	0.0134 (10)	0.0088 (11)
C14	0.0691 (14)	0.0706 (14)	0.0398 (10)	−0.0091 (11)	0.0204 (10)	−0.0092 (10)

### Geometric parameters (Å, °)

**Table:**

N1—C9	1.380 (2)	C5—H5	0.98 (2)
N1—C1	1.393 (2)	C6—C7	1.445 (2)
N1—C14	1.463 (3)	C7—C8	1.360 (2)
O1—C9	1.250 (2)	C8—C9	1.437 (2)
O2—C7	1.341 (2)	C8—C10	1.507 (2)
O2—H2O	0.95 (3)	C10—C11	1.504 (2)
O3—C11	1.201 (2)	C10—H10B	1.021 (18)
O4—C11	1.335 (2)	C10—H10A	0.935 (19)
O4—C12	1.453 (2)	C12—C13	1.507 (3)
C1—C6	1.393 (2)	C12—H12B	0.95 (2)
C1—C2	1.414 (2)	C12—H12A	1.02 (3)
C2—C3	1.377 (3)	C13—H13C	1.05 (4)
C2—H2	0.92 (3)	C13—H13B	0.98 (3)
C3—C4	1.378 (3)	C13—H13A	0.94 (3)
C3—H3	0.99 (3)	C14—H14C	1.00 (3)
C4—C5	1.379 (3)	C14—H14B	0.95 (3)
C4—H4	0.97 (3)	C14—H14A	0.98 (3)
C5—C6	1.409 (2)		
			
C9—N1—C1	121.71 (14)	O1—C9—C8	122.39 (14)
C9—N1—C14	117.82 (16)	N1—C9—C8	118.67 (14)
C1—N1—C14	120.46 (16)	C11—C10—C8	114.00 (13)
C7—O2—H2O	118.5 (15)	C11—C10—H10B	109.2 (10)
C11—O4—C12	117.13 (15)	C8—C10—H10B	108.6 (10)
C6—C1—N1	120.06 (14)	C11—C10—H10A	106.7 (11)
C6—C1—C2	118.72 (17)	C8—C10—H10A	110.0 (10)
N1—C1—C2	121.21 (17)	H10B—C10—H10A	108.2 (14)
C3—C2—C1	120.0 (2)	O3—C11—O4	123.65 (16)
C3—C2—H2	123.1 (16)	O3—C11—C10	125.92 (15)
C1—C2—H2	116.8 (16)	O4—C11—C10	110.43 (14)
C2—C3—C4	121.30 (18)	O4—C12—C13	106.40 (19)
C2—C3—H3	122.3 (17)	O4—C12—H12B	108.5 (15)
C4—C3—H3	116.4 (16)	C13—C12—H12B	112.4 (14)
C3—C4—C5	119.7 (2)	O4—C12—H12A	108.7 (14)
C3—C4—H4	124.4 (15)	C13—C12—H12A	111.0 (13)
C5—C4—H4	115.9 (16)	H12B—C12—H12A	110 (2)
C4—C5—C6	120.3 (2)	C12—C13—H13C	113.3 (18)
C4—C5—H5	122.7 (11)	C12—C13—H13B	110 (2)
C6—C5—H5	117.1 (11)	H13C—C13—H13B	114 (3)
C1—C6—C5	119.99 (15)	C12—C13—H13A	106.8 (18)
C1—C6—C7	118.66 (14)	H13C—C13—H13A	105 (3)
C5—C6—C7	121.36 (17)	H13B—C13—H13A	107 (3)
O2—C7—C8	124.99 (14)	N1—C14—H14C	107.4 (15)
O2—C7—C6	114.46 (14)	N1—C14—H14B	106.9 (18)
C8—C7—C6	120.53 (15)	H14C—C14—H14B	112 (2)
C7—C8—C9	120.23 (14)	N1—C14—H14A	108.7 (15)
C7—C8—C10	122.64 (15)	H14C—C14—H14A	107.6 (19)
C9—C8—C10	117.11 (14)	H14B—C14—H14A	114 (2)
O1—C9—N1	118.92 (15)		
			
C9—N1—C1—C6	3.6 (2)	O2—C7—C8—C9	178.40 (14)
C14—N1—C1—C6	−177.17 (16)	C6—C7—C8—C9	−0.4 (2)
C9—N1—C1—C2	−175.56 (14)	O2—C7—C8—C10	−0.3 (2)
C14—N1—C1—C2	3.7 (2)	C6—C7—C8—C10	−179.14 (14)
C6—C1—C2—C3	0.5 (2)	C1—N1—C9—O1	176.58 (14)
N1—C1—C2—C3	179.68 (15)	C14—N1—C9—O1	−2.7 (2)
C1—C2—C3—C4	1.1 (3)	C1—N1—C9—C8	−4.7 (2)
C2—C3—C4—C5	−1.5 (3)	C14—N1—C9—C8	176.02 (16)
C3—C4—C5—C6	0.2 (3)	C7—C8—C9—O1	−178.23 (15)
N1—C1—C6—C5	179.10 (14)	C10—C8—C9—O1	0.5 (2)
C2—C1—C6—C5	−1.7 (2)	C7—C8—C9—N1	3.1 (2)
N1—C1—C6—C7	−0.8 (2)	C10—C8—C9—N1	−178.10 (13)
C2—C1—C6—C7	178.41 (14)	C7—C8—C10—C11	90.75 (19)
C4—C5—C6—C1	1.4 (3)	C9—C8—C10—C11	−87.98 (19)
C4—C5—C6—C7	−178.75 (16)	C12—O4—C11—O3	−1.4 (2)
C1—C6—C7—O2	−179.69 (13)	C12—O4—C11—C10	178.93 (15)
C5—C6—C7—O2	0.4 (2)	C8—C10—C11—O3	7.3 (3)
C1—C6—C7—C8	−0.7 (2)	C8—C10—C11—O4	−173.09 (13)
C5—C6—C7—C8	179.38 (15)	C11—O4—C12—C13	−175.83 (16)

### Hydrogen-bond geometry (Å, °)

**Table:**

*D*—H···*A*	*D*—H	H···*A*	*D*···*A*	*D*—H···*A*
O2—H2O···O1^i^	0.95 (3)	1.71 (3)	2.649 (2)	169 (2)
C10—H10a···O1^i^	0.94 (2)	2.34 (3)	3.235 (2)	159 (2)

Symmetry codes: (i) −*x*+1/2, *y*+1/2, −*z*+1/2.
